# Essential Fatty Acid Deficiency Associates with Growth Faltering and Environmental Enteric Dysfunction in Children

**DOI:** 10.3390/metabo13040489

**Published:** 2023-03-29

**Authors:** Monica Narvaez-Rivas, Kenneth D. R. Setchell, Stephanie L. Galandi, Xueheng Zhao, Najeeha Talat Iqbal, Sheraz Ahmed, Junaid Iqbal, Sana Syed, Syed Asad Ali, Sean R. Moore

**Affiliations:** 1 Division of Pathology & Laboratory Medicine, Cincinnati Children’s Hospital Medical Center, Cincinnati, OH 45229, USA; monica.narvaezrivas@cchmc.org (M.N.-R.); xueheng.zhao@cchmc.org (X.Z.); 2 Department of Pediatrics, University of Cincinnati College of Medicine, Cincinnati, OH 45229, USA; 3 Departments of Pediatrics and Child Health, Biological and Biomedical Sciences, Aga Khan University, Karachi 74800, Pakistan; 4 Departments of Pediatrics and Child Health, Aga Khan University, Karachi 74800, Pakistan; 5 Division of Pediatric Gastroenterology, Hepatology, and Nutrition, Department of Pediatrics, University of Virginia, Charlottesville, VA 22903, USA

**Keywords:** environmental enteric dysfunction (EED), biomarkers, non-esterified fatty acids (NEFA), stunting, undernutrition

## Abstract

Environmental enteric dysfunction (EED) is characterized by intestinal inflammation, malabsorption and growth-faltering in children with heightened exposure to gut pathogens. The aim of this study was to characterize serum non-esterified fatty acids (NEFA), in association with childhood undernutrition and EED, as potential biomarkers to predict growth outcomes. The study comprised a cohort of undernourished rural Pakistani infants (*n* = 365) and age-matched controls followed prospectively up to 24 months of age. Serum NEFA were quantified at ages 3–6 and 9 months and correlated with growth outcomes, serum bile acids and EED histopathological biomarkers. Serum NEFA correlated with linear growth-faltering and systemic and gut biomarkers of EED. Undernourished children exhibited essential fatty acid deficiency (EFAD), with low levels of linoleic acid and total n-6 polyunsaturated fatty acids, compensated by increased levels of oleic acid and increased elongase and desaturase activities. EFAD correlated with reduced anthropometric Z scores at 3–6 and 9 months of age. Serum NEFA also correlated with elevated BA and liver dysfunction. Essential fatty acid depletion and altered NEFA metabolism were highly prevalent and associated with acute and chronic growth-faltering in EED. The finding suggests that targeting early interventions to correct EFAD and promote FA absorption in children with EED may facilitate childhood growth in high-risk settings.

## 1. Introduction

Childhood undernutrition remains one of the most important global public health challenges of our time. Undernutrition affects ~51 million children under the age of 5 years and is a leading cause of childhood deaths [1,2]. Undernutrition and exposure over time to poor sanitation and hygiene are frequently linked with environmental enteropathy (EE), also known as environmental enteric dysfunction (EED) [3]. EED is characterized clinically by small intestine villous blunting and inflammation [4]. These structural changes promote functional changes, including gut barrier disruption, chronic inflammation, impaired gut immune function and oral vaccine failure [5]. All these changes lead to reduced nutrient uptake and may negatively affect nutrient absorption, consequently worsening the undernutrition state and affecting growth and development [3,4,5].

Altered lipid metabolism is an important component of undernutrition [6,7,8]. Deficiencies in essential and long chain fatty acids (FA) are seen in undernourished children, not only because of reduced dietary intake, poor digestion and absorption of lipids but also subsequent to changes in the hepatic chain elongation and desaturation enzyme systems [8]. Few studies have focused on the FA status of malnourished children [6,7,9,10,11,12,13,14], and most involve small numbers of subjects. Generally, undernourished children display decreased plasma total lipid linoleic acid and its metabolites, with associated increases in the saturated and non-essential monoenoic FA [10,12,14]. The severity of undernutrition has been linked with an increase in desaturation and elongation of polyunsaturated fatty acids (PUFA) and essential FA deficiency, and/or peroxidation [6]. Most studies have focused on total lipid FA in plasma, serum and erythrocytes, and none have studied non-esterified fatty acids (NEFAs). The NEFAs are lipid species that are released from adipocytes and several cell types during lipolysis, transported in the plasma as a complex with albumin, and are a major source of fatty acids for tissues [15]. These NEFAs are not only essential as energy sources but also function as signaling molecules that regulate several cellular and physiological functions depending on their carbon chain length [16]. High concentrations of NEFA in serum have been described in several metabolic disorders and in immune-mediated diseases [17].

The objectives of this study were to better understand lipid dysfunction during malnutrition in the context of the serum non-esterified fatty acid composition of young undernourished rural Pakistani children enrolled in a large Study of Environmental Enteropathy and Malnutrition (SEEM) [18,19,20] by exploring the association between essential fatty acid profiles and growth outcome over 2 years. Furthermore, given the importance of bile acids in lipid digestion and the previously reported finding in this same cohort in which the bile acid metabolome revealed a high rate of subclinical cholestasis with specific bile acid biomarkers predictive of later growth outcomes [19], we aimed to determine the relationship between NEFA and bile acid composition.

## 2. Materials and Methods

### 2.1. Study Design and Specimen Collection

This study of environmental enteropathy and malnutrition (SEEM) is a large prospective study that enrolled children at birth in the rural district of Matiari, Pakistan, between 2016 and 2019 [2]. This trial was registered at ClinicalTrials.gov with Identifier NCT03588013.

The SEEM cohort included a control group of well-nourished children (*n* = 51) and a group of undernourished children (*n* = 365) that were thoroughly evaluated for symptoms of EED at the time of enrollment and were subsequently followed for growth up to 24 months of age. Undernourished children were defined by weight-for-height Z score (WHZ) < −2 at the time of enrollment, and the well-nourished control group was based on a consistent WHZ > 0 and height-for-age Z score (HAZ) > −1 on 2 consecutive visits between 3–6 months of age. Linear and ponderal growth for each child were checked monthly. Blood samples were collected at 3–6 months and 9 months of age, respectively. After enrollment, the parents/caregivers of all participants were given a series of educational programs focused on breast-feeding and complimentary feeding to enhance the child’s nutritional status. If the WHZ score remained < −2 by 9 months of age despite the initial educational counseling, a nutritional intervention was given to the child (*n* = 189) consisting of high calorie Acha Mum supplementary food containing protein and essential fatty acids (World Food Programme, Pakistan). Those undernourished children (*n* = 63) who responded poorly to the nutritional intervention were evaluated by esophagogastroduodenoscopy (EGD) at the Aga Khan University (AKU) hospital. Biopsy specimens were obtained for detailed assessment of histopathology of EED. A subset of children that responded poorly to nutritional intervention declined EGD (*n* = 8). The detailed study design and sample collection timeline were previously described [19] and can be found in Supplementary Figure S1.

### 2.2. NEFA Profiling by Gas Chromatography Coupled with Flame Ionization Detection (GC-FID)

Individual NEFA levels were analyzed in serum following the method previously described by Lepage and Roy [20] where the NEFAs were analyzed as fatty acid methyl esters (FAMEs) by GC-FID [21]. The extraction was carried out in a glass tube, adding 75 µL of human serum to 10 µg of internal standard (tridecanoic acid, C13:0) which was dissolved in 5 mL of methanol-acetyl chloride 50:1 (*v*/*v*). The incubation was held for 45 min at 24–29 °C in a heating block. The methylation reaction was stopped with 3 mL of sodium carbonate 6%. FAMEs were extracted with 150 µL of hexane and placed in an injection vial for their analysis by GC. The FAME profiling was performed using an Agilent 7890 GC system (Agilent Technologies, Santa Clara, CA, USA) coupled to a flame ion detector (FID). A HP-88 column (100 m × 0.25 mm) with a film thickness of 0.2 μm (Agilent Technologies) was used for separation of FAMEs. One microliter of sample was injected using split mode (ratio 1/5) into the GC-FID inlet, which was held at a temperature of 250 °C. The oven temperature was kept at 100 °C for 3 min and then increased to 175 °C at a rate of 8 °C min^−1^, followed by 3 °C min^−1^ up to 240 °C and held isothermally for 10.0 min. Helium was used as carrier gas and the flow rate was 2 mL min−1. While the detector temperature was 240 °C, air and hydrogen with flow rates of 450 and 40 mL min^−1^, respectively, were used for the detector, which had a makeup flow of helium at 25 mL min^−1^.

Quantification was performed using tridecanoic acid, C13:0, as internal standard and interpolation of individual calibration curves for each NEFA. Concentrations were expressed as µg/mL of serum. A total of 31 non-esterified fatty acids were accurately measured, which included decanoic acid (C10:0), lauric acid (C12:0), myristic acid (C14:0), myristoleic acid (C14:1n-5), pentadecanoic acid (C15:0), palmitic acid (C16:0), palmitoleic acid (C16:1n-7), heptadecanoic acid (C17:0), heptadecenoic acid (C17:1n-7), stearic acid (C18:0), oleic acid (C18:1n-9 cis), vaccenic acid (C18:1n-7), linoleic acid (C18:2n-6 cis, LA), α-linolenic acid (C18:3n-3, ALA), γ-linolenic acid (C18:3n-6, GLA), arachidic acid (C20:0), eicosenoic acid (C20:1n-9), eicosadienoic acid (C20:2n-6), 11,14,17-eicosatrienoic acid (C20:3n-3), dihomo-γ-linolenic acid (C20:3n-6, DGLA), arachidonic acid (C20:4n-6, ARA), eicosapentaenoic acid (C20:5n-3, EPA), behenic acid (C22:0), 13,16-docosadienoic acid (C22:2n-6), 13,16,19-docosatrienoic acid (C22:3n-3), docosatetraenoic acid (C22:4n-6), docosapentaenoic acid n-3 (C22:5n-3, DPA n-3), docosapentaenoic acid n-6 (C22:5n-6, DPA n-6), docosahexaenoic acid (C22:6n-3, DHA), tetracosanoic acid (C24:0) and hexacosanoic acid (C26:0).

Calibration curves for all fatty acids were linear over a concentration range of 0.5–500 μg/mL, being the coefficient of determination (R2) for all analytes greater than 0.99. The precisions and accuracies were evaluated with quality control (QC) standards at low (LQC, 0.5 μg/mL), middle (MQC, 125 μg/mL) and high (HOC, 250 μg/mL) concentrations covering analytical ranges. All fatty acids at LQC, MQC and HQC exhibited acceptable accuracies (80–110%). The precisions at the three levels were all below 15%. The lower limit of quantification (LLOQ) for all the fatty acids fell at 0.5 μg/mL, and the lower limit of detection (LLOD) for all the fatty acids fell between 0.15 and 0.25 μg/mL.

All samples were analyzed at a single center, Cincinnati Children’s Hospital Medical Center (Cincinnati, OH, USA). The facility is accredited with the College of American Pathologists (CAP license #1667801) and with Clinical Laboratory Improvement Amendments certification (CLIA 88 license #36D0656333).

Serum bile acid concentrations and composition were measured by tandem mass spectrometry in this cohort as previously reported in detail for this cohort [19].

### 2.3. Investigated Clinical Biomarkers of EED

Blood Cytokine/Chemokine assays used the MILLIPLEX MAP Human Cytokine/Chemokine panel (EMD Millipore corporation, Billerica, MA, USA). α-1 acid glycoprotein (AGP), ferritin and C- reactive protein (CRP) were analyzed using an automated biochemistry analyzer, Roche/Hitachi 902 (Basel, Switzerland), and insulin-like growth factor 1 (IGF-I) was analyzed on a LIAISON Diasorin (Saluggia, Italy) as described previously [18,19]. Mean values and 95% confidence intervals (CI) for all the investigated EED biomarkers of Pakistani undernourished and healthy children at the 2 study time points, 3–6 and 9 months of age, were previously described in detail [19].

### 2.4. Statistical Analysis

Precise comparisons between the well-nourished controls and undernourished groups were evaluated with Student’s t-tests or Welch’s t-test, depending on variance homogeneity. If the ratio of the larger variance to the smaller variance was less than 4, we assumed variances were approximately equal, and the Student’s *t*-test was used. If the ratio between variances was higher than 4, the variances were assumed to be unequal, and the Welch’s t-test was applied. A value of *p* < 0.05 was considered statistically significant. Due to the exploratory nature of the study, multiple testing corrections were not performed. For exploratory analysis, Pearson’s correlation was conducted to elucidate the associations between NEFA composition in the serum and Z scores. For correlation between NEFA and total serum bile acids, a Random Forest analysis was performed to assess the variable importance based on accuracy, followed by a Pearson’s correlation for linear prediction. Dispersion of data was reported as mean ± the standard error of the mean (SEM) or 95% CI unless indicated otherwise. All statistical analysis was conducted using OriginPro 9.1 (OriginLab Corporation, Northampton, MA, USA) and the MetaboAnalyst 5.0 web platform (www.metaboanalyst.ca, accessed on 1 February 2023).

## 3. Results

### 3.1. Summary of the Study Cohorts

The demographics and anthropometrics of the two groups, undernourished and nourished controls, are summarized in Table 1.

### 3.2. Serum NEFA Profiles at 3–6 and 9 Months of Age in Pakistan Children

Serum NEFA profiles, including saturated and unsaturated species of 10 to 26 carbon chain lengths were quantified at both 3–6 and 9 months of age and prior to nutritional intervention in order to study the association between NEFA profiles and nutritional status. A total of 724 serum samples were analyzed from children over these 2 time points. A total of 678 serum samples were paired for both age groups. To further understand the metabolic differences between undernourished and healthy controls, the concentration and percent composition of 31 NEFA species were analyzed using the corresponding t-test analysis at both time points. Four of the 31 NEFAs, C22:0, C22:2n-6, C24:0 and C26:0, were present at extremely low concentrations or were undetectable in multiple samples but nevertheless were included in the data analysis. The Mead acid (C20:3n-9), which has been used as an indicator of essential fatty acid deficiency in some studies [8,10], was detectable but below the lower limit of quantification (LLOQ) of the assay. This may be because of the early age of the children. Interestingly, C18:2n-3 levels were not drastically deficient, which would be consistent with the failure to find increased levels of the Mead acid (C20:3n-9) because an increase in the latter would be expected if both C18:2n-6 and C18:3n-3 were deficient [8].

**Figure 1:**
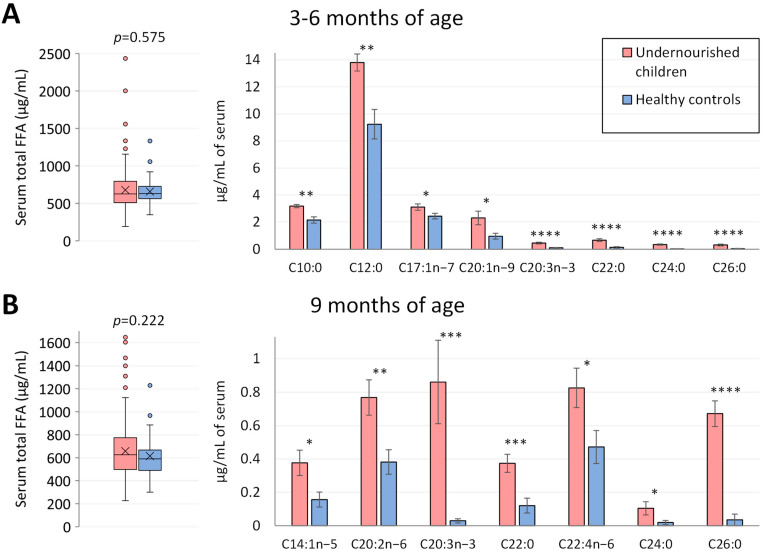
Serum NEFA profiles in Pakistani undernourished and healthy children at 3–6 months and 9 months of age. (**A**,**B**) Serum NEFA profile in Pakistani undernourished children and healthy controls at 3–6 (*n* = 335 undernourished and 50 healthy controls) and 9 months of age (*n* = 292 undernourished and 47 healthy controls). On the left, total non-esterified fatty acid profiles were plotted, and on the right, individual NEFA that showed significant differences between the two groups. * *p* < 0.05, ** *p* < 0.01, *** *p* < 0.001, **** *p* < 0.0001. NEFA, non-esterified fatty acid; C10:0, decanoic acid; C12:0, lauric acid; C14:1n-9, myristoleic acid; C17:1n-7, heptadecenoic acid; C20:1n-9, eicosenoic acid; C20:2n-6, eicosadienoic acid; C20:3n-3, 11,14,17-eicosatrienoic acid; C22:0, behenic acid; C22:4n-6, docosatetraenoic acid; C24:0, tetracosanoic acid; C26:0, hexacosanoic acid.

Measurements of total serum NEFA concentrations did not show statistically significant differences between undernourished children and healthy controls at age 3–6 months (*p* = 0.575) or at age 9 months (*p* = 0.222) (Figure 1). However, when individual NEFA profiles were examined in more detail, significant differences were found for 8 NEFA species at 3–6 months of age and for 7 NEFA species at 9 months of age. At both time points, in the undernourished group, the concentrations of C20:3n-3, C22:0, C24:0 and C26:0 were at least 4.5-fold greater than in the local Pakistani control group. Specifically at 3–6 months (Figure 1A), the concentrations of C10:0 (fold-change = 1.5, *p* < 0.01), C12:0 (fold-change = 1.5, *p* < 0.01), C17:1n-7 (fold-change = 1.3, *p* < 0.05) and C20:1n-9 (fold change = 2.4, *p* < 0.05) were significantly higher in undernourished cases when compared to well-nourished controls. At the age of 9 months (Figure 1B), undernourished children presented with significantly higher serum concentrations of free C14:1n-5 (fold-change = 2.4, *p* < 0.05), C20:2n-6 (fold-change = 2.0, *p* < 0.01) and C22:4n-6 (fold-change = 1.7, *p* < 0.05).

**Figure 2:**
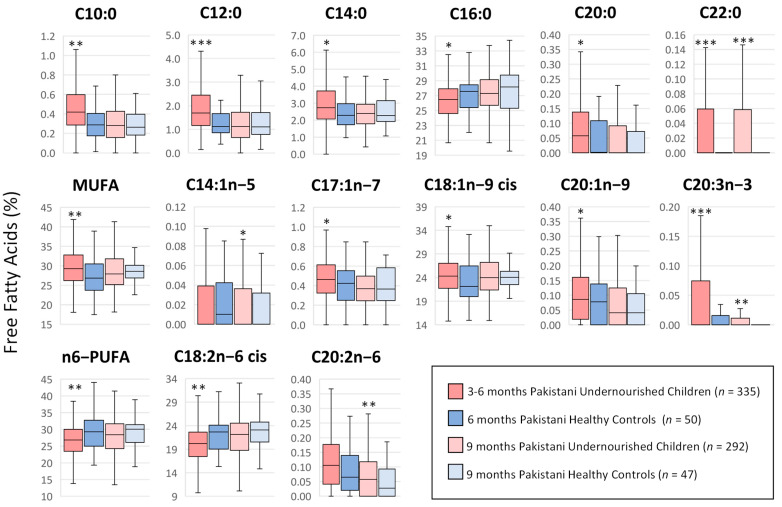
Proportions (%) of serum non-esterified fatty acids in Pakistani undernourished and healthy children at two study time points. Median values and interquartile ranges are shown in boxplots. Differences in each characteristic were evaluated by corresponding *t*-test. *** *p* < 0.0001 compared with the healthy control group at the corresponding time point. ** *p* < 0.01 compared with the healthy control group at the corresponding time point. Median values and interquartile ranges are shown in boxplots. * *p* < 0.05 compared with the healthy control group at the corresponding time point. More specific information is included in Supplementary Table S1.

When percentages of NEFA were considered (Figure 2 and Supplementary Table S1), the most abundant NEFA for both groups at both time points were C16:0, C18:1n-9 cis and C18:2n-6 cis. At the age of 3–6 months, undernourished children exhibited a significantly higher proportion of 7 of the saturated fatty acids (C10:0, C12:0, C14:0, C20:0, C22:0, C24:0 and C26:0) when compared with healthy controls and a lower percentage of C16:0 (26.35 ± 0.15% vs. 27.18 ± 0.37%, *p* < 0.05). However, the proportion of total saturated fatty acid (SFA) was not significantly different between the two groups. For total monounsaturated fatty acids (MUFA), undernourished children had higher proportions (29.73 ± 0.28% vs. 27.71 ± 0.71%, *p* < 0.01), accounted for by higher percentages of C17:1n-7, C18:1n-9cis and C20:1n-9. One of the less abundant n3-PUFA, C20:3n-3, was also significantly higher in the undernourished group (0.063 + 0.011% vs. 0.013 + 0.004%, *p* < 0.0001). On the other hand, one of the most abundant NEFAs and a precursor of essential fatty acids, C18:2n-6 cis, was proportionally lower in undernourished children compared to the well-nourished controls (20.23 ± 0.24% vs. 22.33 ± 0.75%, *p* < 0.01), which contributed to a significantly lower percentage of total n6-PUFA (26.82 ± 0.29% vs. 29.24 ± 0.93%, *p* < 0.01). At 9 months, only C22:0, C26:0, C14:1n-5, C20:3n-3 and C20:2n-6 showed significant differences from controls and resulted in higher proportions in the undernourished group. The totals were comparable between both groups.

**Figure 3:**
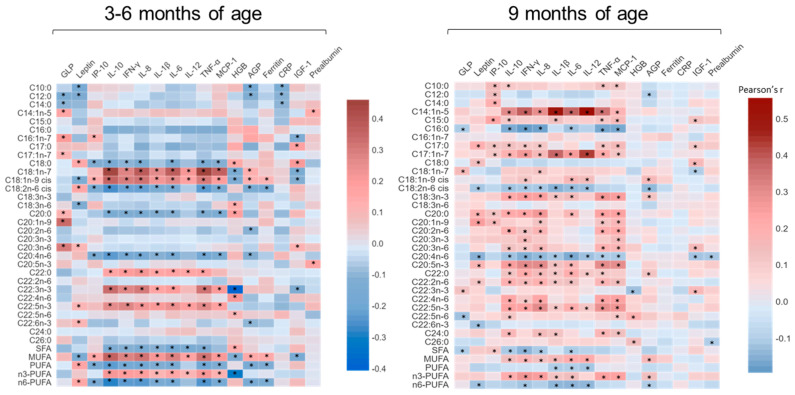
Correlations between NEFAs (%) and cytokines and other EED biomarkers in Pakistani undernourished and healthy children aged 3–6 months and at 9 months. AGP, α−1 acid glycoprotein; CRP, C−reactive protein; EED, environmental enteric dysfunction; GLP, glucagon−like peptide 2; HGB, hemoglobin; IGF−1, insulin-like growth factor 1; IL−8, interleukin 8; IL−6, interleukin 6; IL−10, interleukin 10; IL−12, interleukin 12; IL−1β, interleukin 1β; INF−γ, interferon−γ; IP−10, interferon-inducible protein 10; MCP−1, monocyte chemoattractant protein 1; TNF−α, Tumor Necrosis Factor alpha; * *p* < 0.05.

Further analysis of the serum NEFA concentrations, when expressed as a percentage of the total NEFAs, showed significant correlations with multiple blood EED biomarkers at the age of 3–6 months and at 9 months (Figure 3). Multiple NEFAs correlated significantly with pro-inflammatory cytokines, including interleukin 1β (IL-1β) and tumor necrosis factor alpha (TNF-α), and anti-inflammatory cytokines, including interleukin 6(IL-6), at both assessed time points. Total SFA, PUFA and n6-PUFA were negatively correlated with cytokine levels, while total MUFA and n3-PUFA were positively correlated at both time points of the study. Similarly, individual proportions of NEFA also correlated with other EED biomarkers, including glucagon-like peptide 2 (GLP), leptin, hemoglobin (HGB), α-1 acid glycoprotein (AGP) and insulin-like growth factor 1 (IGF-1). These results support the notion that NEFA can modulate the production of chemokines and cytokines and give rise to pro-inflammatory and inflammation pro-resolving lipid derived species [22], making them potential biomarkers for EED prediction.

### 3.3. Enzyme Activity Indices Using Product/Precursor Ratios

**Figure 4:**
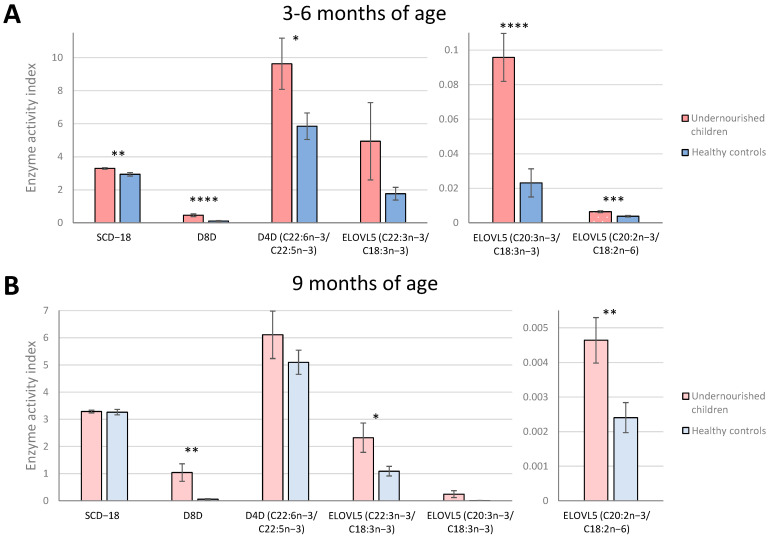
Markers of desaturase and elongase indices associated with malnutrition status at both time points. Enzyme activity indices estimated by fatty acid ratios in Pakistani undernourished children and healthy controls at age 3–6 months (**A**) and 9 months (**B**). Fatty acid unit of measurement expressed as µg/mL of serum. Bars and error bars represent mean and ± SEM. To compare mean ratio markers between undernourished children and healthy controls, *t*−test was used. * *p* < 0.05, ** *p* < 0.01, *** *p* < 0.001, **** *p* < 0.0001. SCD-18, stearoyl−CoA desaturase−18; D8D, delta−8−desaturase; D4D, delta−4-desaturase; ELOVL5, elongase 5.

**Figure 5:**
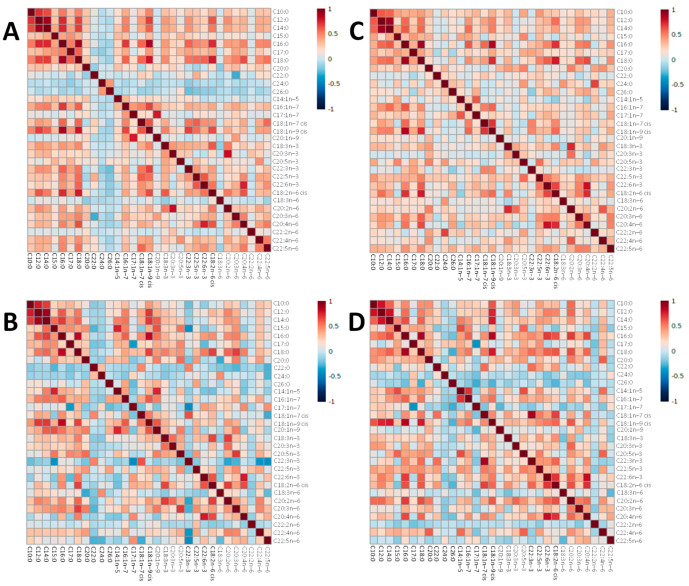
Heatmaps representing pairwise correlations between all serum non-esterified fatty acids concetrations (ug/mL) in undernourished and healthy control children at 3–6 months and 9 months of age. In the heatmap, red represents higher correlation, and blue shows lower correlation between a pair of markers. (**A**) Undernourished group at 3–6 months. (**B**) Healthy control group at 3–6 months. (**C**) Undernourished group at 9 months. (**D**) Healthy control group at 9 months.

Product/precursor ratios of fatty acids were used as proxy markers of enzyme activities for stearoyl-CoA desaturase-16 and 18 (SCD-16 and SCD-18), delta-8-desaturase (D8D), delta-6-desaturase (D6D), delta-5-desaturase (D5D), delta-4-desaturase (D4D) and elongases 5 and 6 (ELOVL5 and ELOVL6) (Table 2, Supplementary Figure S2). These ratios usually facilitate an estimate of the in vivo activity of these enzymes and serve to help explore whether a FA regulatory enzyme contributes to FA differences [23]. At 3–6 months of age, 5 proxy markers were significantly different between the undernourished children and the Pakistani well-nourished controls (Figure 4A). The markers for SCD-18, D8D, D4D (C22:6n-3/C22:5n-3) and two markers for ELOVL5 (C20:3n-3/C18:3n-3 and C20:2n-6/C18:2n-6) were increased in undernourished children compared with healthy controls. At the age of 9 months (Figure 4B), only 3 markers presented significant differences: D8D (fold change = 17.9, *p* < 0.01) and two markers for ELOVL5 (C22:3n-3/C18:3n-3, fold change = 2.1, *p* < 0.05; and C20:2n-6/C18:2n-6, fold change = 1.9, *p* < 0.01), being significantly higher for the undernourished group. Two of these, D8D and ELOVL5 (C20:2n-6/C18:2n-6), were also higher at the age of 3–6 months. Correlations (Figure 5) between products and precursors for the different enzymes also showed some differences in NEFA concentrations between undernourished children and healthy controls at both time points, establishing that FA metabolism is clearly altered by malnutrition.

### 3.4. Correlations between Non-Esterified Fatty Acid Concentrations and Growth Outcomes

Concentrations of serum NEFA were correlated with the different Z scores, HAZ, WHZ and HAZ at 3–6 and 9 months of age (Supplementary Table S2). WHZ negatively correlated with C10:0 and C12:0 at 3–6 months and positively with C20:1n-9 at 9 months. More significant correlations were observed between NEFA concentrations and HAZ and WAZ. At age 3–6 months, 5 NEFA were significantly negatively correlated with HAZ and 6 with WAZ, while only C20:4n-6 and C22:6n-3 showed a positive correlation with HAZ and WAZ. It was noticeable that similar negative correlations for HAZ and WAZ were observed for C12:0, C14:0, C18:3n-6, C20:3n-3, C20:4n-6, C22:6n-3 and C24:0. At 9 months of age, 3 NEFA and total PUFA and total n6-PUFA showed a significant positive correlation with HAZ, while only C18:1n-7 showed a negative correlation. No NEFA correlated with WAZ at 9 months.

More significant correlations were found between the different Z scores and the relative proportion of individual NEFA species when expressed as a percentage of the total serum NEFA in samples at 3–6 and 9 months of age and prior to any nutritional intervention (Table 3 and Supplementary Figure S3). Fourteen NEFA correlated with at least one of the Z scores, in addition to total MUFAs, PUFAs and n6-PUFAs. Of note, C18:1n-9 cis and total MUFAs were significantly negatively correlated to HAZ score at both time points, while a significant positive correlation was observed for C20:4n-6 and C22:6n-3. The same trend was observed for these last two species with the serum concentrations (µg/mL) indicating that these NEFA may be potential biomarkers for early growth-faltering.

**Figure 6:**
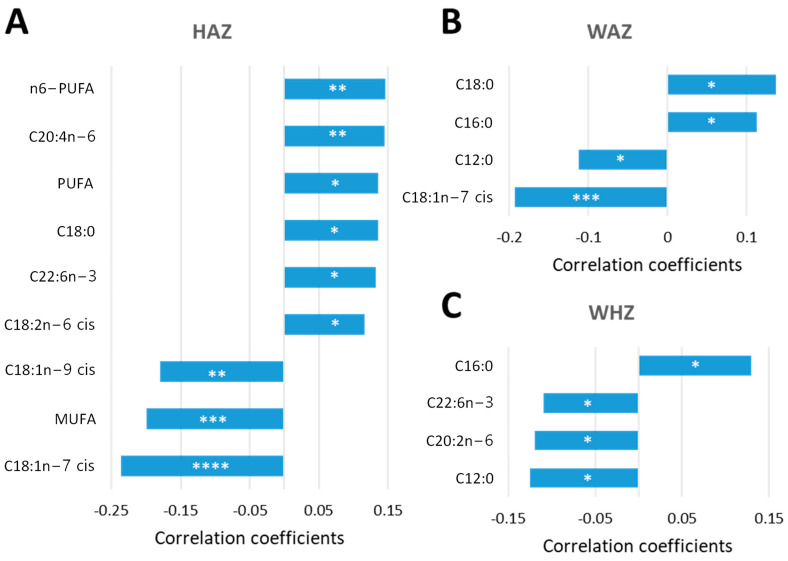
NEFA biomarkers as predictors of long-term growth outcome. Percentages of NEFAs at 9 months of age (data set consisted of 322 Pakistani children) were correlated with HAZ (**A**), WAZ (**B**) and WHZ (**C**) scores at 24 months using a pattern search and Pearson’s r as distant measure. Significant linear correlations are considered when the *p*-value was lower than 0.05. * *p* < 0.05, ** *p* < 0.01, *** *p* < 0.001, **** *p* < 0.0001.

In order to identify potential predictive relationships between NEFA biomarkers and acute and long-term growth outcomes at 24 months of age, individual NEFA species were correlated with HAZ, WHZ and WAZ scores. Percentages were chosen over concentrations at this point due to a higher number of linear correlations found, as previously described. Using Pearson’s r as distant measure, the correlation analysis performed showed that multiple NEFA biomarkers at 9 months of age expressed as a percentage of the total serum non-esterified fatty acids were predictive of linear growth at 24 months of age (Figure 6). Among these NEFA species, C20:4n-6, C18:0, C22:6n-3 and C18:2n-6 cis, along n6-PUFA and PUFA, positively correlated with HAZ scores at 24 months, while C18:1n-9 cis, C18:1n-7 and MUFA were negatively correlated (Figure 6A). Remarkably, lower HAZ scores were correlated with lower amounts of C18:2n-6 cis and n6-PUFA and higher percentages of C18:1n-9, supporting essential fatty acid deficiency with subsequent weight and height retardation [24]. Two saturated NEFA (C16:0 and C18:0) were positively associated with WAZ score, while C12:0 and C18:1n-7 showed an inverse relationship (Figure 6B). Serum C16:0 also correlated positively with WHZ, while a negative correlation was observed for this Z score with C12:0, C20:2n-6 and C22:6n-3 (Figure 6C). Thus, at 9 months of age, NEFA biomarkers significantly correlated with linear growth of the child at 24 months of age, suggesting these may serve as potential biomarkers for predicting growth outcomes.

### 3.5. Non-Esterified Fatty Acids and Cholestasis

**Figure 7:**
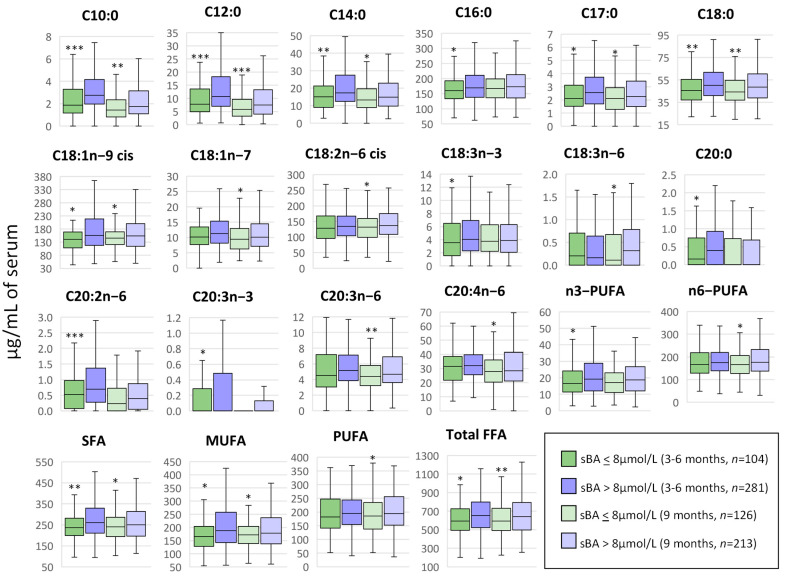
Correlation between serum NEFA and bile acids in Pakistani children at 3–6 months and 9 months of age. At both time points, concentrations of serum NEFAs (µg/mL) were correlated with total serum bile acid (sBA) concentration (µmol/L) by using a t-test, where the grouping criteria considered were sBA < 8 µmol/L (normal concentration) and sBA > 8 µmol/L (liver dysfunction and/or cholestasis). Median values and interquartile ranges are shown in boxplots. * *p* < 0.05, ** *p* < 0.01, *** *p* < 0.001. NEFA, non-esterified fatty acid; C10:0, decanoic acid; C12:0, lauric acid; C14:0, myristic acid; C16:0, palmitic acid; C17:0, heptadecanoic acid; C18:0, stearic acid; C18:1n-9 cis, oleic acid; C18:1n-7, vaccenic acid; C18:2n-6 cis, linoleic acid; C18:3n-3, α-linolenic acid; C18:3n-6, γ-linolenic acid; C20:0, arachidic acid; C20:2n-6, eicosadienoic acid; C20:3n-3, 11,14,17-eicosatrienoic acid; C20:3n-6, dihomo-γ-linolenic acid; C20:4n-6, arachidonic acid; SFA, total saturated fatty acids; MUFA, total monounsaturated fatty acids; PUFA, total polyunsaturated fatty acids; n3-PUFA, total n-3 polyunsaturated fatty acids; n6-PUFA, total n-6 polyunsaturated fatty acids.

Total and individual serum bile acid (sBA) concentrations for these patients were previously published by our group [19] and revealed that total sBA concentrations in 75% of the undernourished children were significantly higher than in the healthy controls at 3–6 months and 64% higher at 9 months of age, consistent with subclinical chronic liver dysfunction and/or cholestasis. Cholestatic liver disease impacts lipid absorption and metabolism, bile acids being crucial to intestinal lipid absorption and essential in preventing nutritional deficiencies and growth complications [25]. We therefore performed a correlation analysis between serum NEFA concentrations and previously determined sBA concentrations at 3–6 months (*n* = 385) and 9 months (*n* = 339) of age (Supplementary Figures S4 and S5). A random forest (RF) analysis was performed to evaluate the relative importance of NEFA in liver dysfunction and/or cholestasis at the ages of 3–6 months (Supplementary Figure S4A) and 9 months (Supplementary Figure S4C) with the threshold of 8 µmol/L being the upper limit of the normal range. Of note, C10:0, C12:0, C17:0, C18:0, C18:1n-9 cis, C18:2n-6 cis, MUFA, PUFA, n6-PUFA and total NEFA were among the top 15 predictors in the RF analysis for both time-points, showing higher concentrations when sBA > 8 µmol/L. In addition to C18:0 and total SFA, 3 of these NEFA (C10:0, C12:0 and C14:0) and the total NEFA concentration were positively and significantly correlated with the total sBA concentration at both 3–6 months (Supplementary Figure S4B) and 9 months (Supplementary Figure S4D) of age. All these species and totals, along with C17:0, C18:1n-9 cis and MUFA, were significantly higher in children with sBA concentrations above the normal range when compared with children with normal levels of sBA at both time points (Figure 7). At the age of 3–6 months, 5 additional NEFAs (C16:0, C18:3n-3, C20:0, C20:2n-6 and C20:3n-3) and total n3-PUFA were significantly higher in the group with elevated serum BA concentrations, while at 9 months of age, the NEFAs that presented significant differences associated with the sBA concentration were C18:1n-7, C18:2n-6 cis, C18:3n-6, C20:3n-6, C20:4n-6, n6-PUFA and total PUFA. It is noteworthy that elevated concentrations of sBA were associated with elevated concentrations of serum NEFAs, which could suggest a disrupted synthesis of apoproteins and other enzymes involved in lipoprotein formation and metabolism and/or an abnormal re-esterification of absorbed fatty acids into triacylglycerols [25].

## 4. Discussion

This manuscript presents a unique investigation of serum non-esterified fatty acid composition, the largest to date in a pediatric cohort, comprising a total of 416 Pakistani children (365 undernourished and 51 age-matched well-nourished controls) who were followed prospectively over 2 years to determine (1) the extent to which fatty acid metabolism is altered in undernourished children and (2) if measures of serum NEFA elucidate the link between EED and adverse growth outcomes in children. A targeted metabolomics approach was used to profile the different saturated and unsaturated NEFA species in serum. Our serial NEFA measurements during the early stage of life in these infants indicate that significant lipid and enzymatic dysregulation were associated with future growth outcomes. Although Total NEFA concentration was consistently elevated in undernourished children when compared with their aged-matched Pakistani well-nourished controls, this difference was not statistically significant. However, significant differences in individual NEFAs were revealed, highlighting the importance of profiling individual NEFA species. Uniquely, this study compared for the first time the relationship between serum NEFA and bile acids and revealed that a high proportion of undernourished children with co-existing liver dysfunction [19] exhibited fatty acid malabsorption and/or alterations in biosynthesis.

NEFAs are lipid species discharged from the adipose tissue and other cell types upon lipolysis. In addition to their conventional roles in energy supply and as structural components, NEFA play a role in numerous biological processes. For example, NEFA regulate gene expression in macrophages, adipocytes and endothelial cells, modulate the production of chemokines and cytokines and the expression of genes coding for adhesion molecules, giving rise to pro-inflammatory and inflammation pro-resolving lipid-derived species [22]. Therefore, NEFA are key mediators between metabolism and the immune system. Further, altered NEFA pool composition has been previously shown to be associated with the risk of developing a range of immune disorders [26,27,28]. EED, a subclinical condition of intestinal inflammation, barrier dysfunction and malabsorption is associated with malnutrition, vaccine failure and neurocognitive development and may also be influenced by disordered NEFA composition [3]. Our findings of impaired serum NEFA profiles in undernourished Pakistani children as young as 3–6 months of age highlights a role of early altered lipid metabolism that may be targetable through maternal nutritional interventions, preventive breastfeeding and complementary lipid-based nutrient supplements. The correlation between serum NEFA and EED biomarkers at 3–6 months and 9 months of age found in this study demonstrates that the NEFA promote pro-inflammatory and inflammation pro-resolving lipid derived species, and that nutritional intervention may help reverse undernutrition via these mechanisms.

The unexpectedly high levels of NEFA found in undernourished Pakistani children may be explained by increased adipose tissue lipolysis promoted by low leptin levels to ensure FA supply as a fuel source for the metabolism in the brain and peripheral tissue and for the maintenance of high cortisol and growth hormone levels during nutritional deprivation [29]. Increased NEFA levels in serum have been described in several metabolic disorders and in immune-mediated diseases [30], and it has been suggested this could be related to changes in the gut ecosystem. NEFA receptors are nutrient sensors expressed in various tissues and cells, regulating energy and immune responses [16]. Changes in nutritional status, as occurs with undernutrition, where the ratios and amounts of NEFA are altered, can disrupt body homeostasis maintained by NEFA receptors, which sense nutritional states and regulate biological processes. This opens the possibility of treating EED patients by targeting and influencing NEFA composition through appropriate high quality fat diets.

Fatty acids are usually classified according to the number of double bonds in the molecule. While saturated fatty acids do not include any double bonds, unsaturated fatty acids include one or more, i.e., monounsaturated and polyunsaturated fatty acids, respectively. Unsaturated fatty acids are classified as n-3, n-6, n-7 and n-9 fatty acids and are found in a selection of foods, including soybeans (n-3), sunflower seed oil (n-6), butter (n-7) and olive and almond oils (n-9) [31]. Due to the absence of certain enzymes in humans (Δ12- and Δ15-desaturases), fatty acids can be endogenously desaturated up to the Δ9 position. Due to this process, linoleic (C18:2n-6 cis, LA) and α-linoleic (C18:3n-3, ALA) acids are considered essential and must be obtained from the diet. These acids are precursors of long-chain polyunsaturated FAs (PUFAs) of the n-3 and n-6 series through further elongation and desaturation in humans, including eicosapentaenoic (C20:5n-3, EPA), docosahexaenoic (C22:6n-3, DHA) and arachidonic (C20:4n-6, AA) acids [31]. The product/substrate FA ratios reflect enzyme activity indices and indirectly permit the evaluation of the activity of several desaturases and elongases. In this regard, our data revealed an upregulation of D8D, D4D and ELOVL5 in undernourished Pakistani children sustained up to the age of 9 months, suggesting the activity of these fatty acid desaturases and elongases may directly contribute to the distinct NEFA signature observed. Previously, high desaturase activities have been described in children with liver steatosis [32] and in undernourished Moroccan children [6]. Further, our data revealed that Pakistani undernourished children exhibited C18:2n-6 cis and total n6-PUFA depletion, with a subsequent compensatory increase in proportion of C18:1n-9 cis, which is a signature of essential fatty acid deficiency [33]. This was more pronounced at the ages of 3–6 months, being evident in 67% of these children but still noticeable at 9 months, with 53% of children affected. Thus, group similarities in NEFA profiles at 9 months of age could be an indication of some success of the nutritional education given to parents earlier. Increases in desaturase activity indices have been found previously in essential fatty acid deficiencies [34,35,36], consistent with our findings.

Many of these children had high total serum bile acid concentrations and low concentrations of the secondary bile acid DCA, indicative of significant cholestasis and liver dysfunction [19], which has also been associated with essential fatty acid depletion [37]. This is confirmed in our study, as elevated concentrations of serum bile acids were strongly associated with elevated concentrations of serum NEFAs. Therefore, it is likely, that disrupted synthesis of apoproteins and other enzymes involved in lipoprotein formation and metabolism and/or an abnormal re-esterification of absorbed fatty acids into triacylglycerols is related to low leptin levels in the undernourished state of these patients. Based on the above-mentioned information, we speculate that increase in lipolysis, alterations in elongases and desaturases and dietary deficiency in essential fatty acids could be underlying factors and markers for the progression of cholestasis.

The finding that the serum NEFA composition at the early age of 9 months correlated with future growth outcome at 24 months shows the importance of recognizing undernutrition early in life [38]. The deficit in essential fatty acids highlighted in this study was associated with linear growth faltering. Various experimental, clinical and epidemiological data have linked conditions in early life to later health outcomes, suggesting that gestation and the first months of life are critical periods of organ development for the fetus and the newborn [39]. Long-chain PUFA are important for fetal growth and development and influence the length of gestation, risk of preeclampsia, preterm birth, low birth weight [40] and promoting appropriate immune development [41].

This was an observational study with several limitations that include the inability to obtain esophagogastroduodenoscopy biopsies in all patients and well-nourished controls for obvious ethical reasons, the lack of defining EED in the early age groups (diagnosis being made only after nutritional failure) and the lack of biochemistries to confirm the presence of cholestasis in a high proportion of the subjects, as this was an unanticipated observation.

## 5. Conclusions

In summary, the key elements and findings from this large longitudinal study of the fatty acid metabolome of undernourished Pakistani children were (1) the finding of early-life specific biomarkers that predict growth outcomes at 2 years of age, (2) findings of an upregulation in desaturases and elongases related to undernutrition, (3) a significant increase in NEFAs in undernourished children, suggesting high lipolysis of the subcutaneous fat associated with low levels of leptin due to a lack of nutrients, (4) a strong correlation between serum NEFA and EED biomarkers and (5) a remarkable correlation between high concentration of serum NEFAs and sub-clinical cholestasis defined from serum bile acid concentrations.

Our study provides evidence that serum non-esterified fatty acids are potential biomarkers for undernutrition, EED pathogenesis and prediction of growth outcomes in infants, suggesting nutritional interventions with the inclusion of essential fatty acids may be beneficial in these children as well as for their mothers during pregnancy or breastfeeding.

## Figures and Tables

**Table 1:** Demographics and anthropometrics ^1^ of Pakistani undernourished and healthy children ^2^.

Demographics and Anthropometrics	Healthy Control (WHZ > 0, HAZ > −1 at Enrollment)		Undernourished Children (WHZ < −2 at Enrollment)	
N	51		365	
Female, %	24 (47%)		142 (39%)	
Age, m	3–6	9	3–6	9
HAZ	−0.665 (−0.951, −0.379)	−0.905 (−1.238, −0.572)	−2.361 (−2.515, −2.207)	−2.504 (−2.664, −2.344)
WAZ	0.699 (0.443, 0.955)	0.638 (0.381, 0.896)	−2.63 (−2.70, −2.56)	−2.04 (−2.16, −1.91)

^1^ Data are represented as mean and 95% CI. ^2^ HAZ, height-for-age Z score; WAZ, weight-for-age Z score; WHZ, weight-for-height Z score.

**Table 2:** Substrates and products of elongases and desaturases investigated in the study.

Enzyme	Substrate	Product	Activity
SCD-16	C16:0	C16:1	
SCD-18	C18:0	C18:1	
D8D	C20:3n-3	C20:4n-3 (ND)	∆8
	C20:2n-6	C20:3n-6	∆8
D6D	C18:3n-3	C18:4n-3 (ND)	∆6
	C18:2n-6	C18:3n-6	∆6
D5D	C20:4n-3 (ND)	C20:5n-3	∆5
	C20:3n-6	C20:4n-6	∆5
D4D	C22:5n-3	C22:6n-3	∆4
	C22:4n-6	C22:5n-6	∆4
ELOVL5	C18:3n-3	C20:3n-3	C18 → 20
		C22:3n-3	C20 → 22
	C18:2n-6	C20:2n-6	C18 → 20
		C22:2n-6	C20 → 22
	C18:3n-6	C20:3n-6	C18 → 20
	C20:5n-3	C22:5n-3	C20 → 22
	C20:4n-6	C22:4n-6	C20 → 22
	C22:5n-3	C24:5n-3 (ND)	C22 → 24
ELOVL6	C16:0	C18:0	C16 → 18

SCD: stearoyl-CoA desaturase, D4D: delta-4-desaturase, D5D: delta-5-desaturase, D6D: delta-6-desaturase, D8D: delta-8-desaturase, ELOVL5: elongase 5, ELOVL6: elongase 6, ND: non detected. More information in Supplementary Figure S2.

**Table 3:** Correlation between serum non-esterified fatty acid biomarkers (expressed as % composition) and growth among Pakistani undernourished and healthy children at 3–6 months and 9 months of age.

NEFA	Age	*n*	WHZ			HAZ			WAZ		
			r	95% CI	*p*-Value	r	95% CI	*p*-Value	r	95% CI	*p*-Value
C10:0	3–6 M	385	−0.162	(−0.258, −0.063)	**0.001**	−0.036	(−0.136, 0.064)	0.484	−0.107	(−0.205, −0.007)	**0.036**
	9 M	339	0.087	(−0.020, 0.192)	0.110	0.032	(−0.075, 0.138)	0.560	0.067	(−0.040, 0.172)	0.219
C12:0	3–6 M	385	−0.164	(−0.260, −0.065)	**0.001**	−0.148	(−0.244, −0.049)	**0.004**	−0.183	(−0.278, −0.085)	**0.0003**
	9 M	339	0.026	(−0.081, 0.132)	0.639	0.024	(−0.083, 0.130)	0.660	0.016	(−0.091, 0.122)	0.770
C14:0	3–6 M	385	−0.080	(−0.179, 0.020)	0.115	−0.134	(−0.231, −0.035)	**0.008**	−0.130	(−0.227, −0.030)	**0.011**
	9 M	339	0.054	(−0.053, 0.160)	0.323	0.002	(−0.105, 0.109)	0.975	0.025	(−0.082, 0.131)	0.648
C16:0	3–6 M	385	0.136	(0.037, 0.233)	**0.008**	0.090	(−0.010, 0.188)	0.076	0.127	(0.027, 0.224)	**0.013**
	9 M	339	0.031	(−0.076, 0.137)	0.569	−0.014	(−0.120, 0.093)	0.803	0.018	(−0.089, 0.124)	0.746
C18:0	3–6 M	385	0.109	(0.009, 0.207)	**0.032**	0.113	(0.013, 0.211)	**0.027**	0.126	(0.026, 0.223)	**0.014**
	9 M	339	−0.007	(−0.113, 0.100)	0.902	0.059	(−0.048, 0.165)	0.278	0.032	(−0.075, 0.138)	0.558
C18:1 *n*-9 cis	3–6 M	385	−0.108	(−0.206, −0.008)	**0.034**	−0.107	(−0.205, −0.007)	**0.037**	−0.137	(−0.234, −0.038)	**0.007**
	9 M	339	−0.018	(−0.124, 0.089)	0.741	−0.190	(−0.291, −0.085)	**0.0004**	−0.122	(−0.226, −0.016)	**0.025**
C18:1 n-7	3–6 M	385	0.001	(−0.099, 0.101)	0.988	−0.039	(−0.138, 0.061)	0.442	−0.030	(−0.130, 0.070)	0.558
	9 M	339	0.011	(−0.096, 0.117)	0.834	−0.176	(−0.277, −0.071)	**0.001**	−0.104	(−0.208, 0.003)	0.056
C18:2 n-6 cis	3–6 M	385	0.134	(0.035, 0.231)	**0.009**	0.068	(−0.032, 0.167)	0.186	0.125	(0.025, 0.222)	**0.014**
	9 M	339	−0.041	(−0.147, 0.066)	0.452	0.146	(0.040, 0.249)	**0.007**	0.059	(−0.048, 0.165)	0.282
C18:3 n-6	3–6 M	385	−0.027	(−0.127, 0.073)	0.596	−0.139	(−0.236, −0.040)	**0.006**	−0.120	(−0.217, −0.020)	**0.019**
	9 M	339	−0.018	(−0.124, 0.089)	0.736	0.033	(−0.074, 0.139)	0.543	0.005	(−0.102, 0.112)	0.934
C20:0	3–6 M	385	−0.110	(−0.208, −0.010)	**0.031**	−0.036	(−0.136, 0.064)	0.477	−0.082	(−0.181, 0.018)	0.108
	9 M	339	0.006	(−0.101, 0.112)	0.906	0.018	(−0.089, 0.124)	0.746	0.011	(−0.096, 0.117)	0.835
C20:1 n-9	3–6 M	385	−0.098	(−0.196, 0.002)	0.055	−0.022	(−0.122, 0.078)	0.666	−0.068	(−0.167, 0.032)	0.182
	9 M	339	0.135	(0.029, 0.238)	**0.013**	−0.017	(−0.123, 0.090)	0.752	0.071	(−0.036, 0.176)	0.194
C20:4 n-6	3–6 M	385	0.047	(−0.053, 0.146)	0.359	0.133	(0.034, 0.230)	**0.009**	0.121	(0.021, 0.218)	**0.017**
	9 M	339	−0.032	(−0.138, 0.075)	0.562	0.171	(0.066, 0.273)	**0.002**	0.084	(−0.023, 0.189)	0.121
C22:6 n-3	3–6 M	385	0.022	(−0.078, 0.122)	0.669	0.218	(0.121, 0.311)	**0.00002**	0.174	(0.075, 0.269)	**0.001**
	9 M	339	−0.030	(−0.136, 0.077)	0.585	0.206	(0.102, 0.306)	**0.0001**	0.105	(−0.002, 0.209)	0.053
C24:0	3–6 M	385	−0.073	(−0.172, 0.027)	0.153	−0.101	(−0.199, −0.001)	**0.048**	−0.110	(−0.208, −0.010)	**0.031**
	9 M	339	0.048	(−0.059, 0.154)	0.375	0.076	(−0.031, 0.181)	0.163	0.074	(−0.033, 0.179)	0.177
MUFA	3–6 M	385	−0.126	(−0.223, −0.026)	**0.014**	−0.104	(−0.202, −0.004)	**0.042**	−0.146	(−0.242, −0.047)	**0.004**
	9 M	339	0.005	(−0.102, 0.112)	0.929	−0.214	(−0.313, −0.110)	**0.0001**	−0.123	(−0.227, −0.017)	**0.024**
PUFA	3–6 M	385	0.094	(−0.006, 0.192)	0.064	0.089	(−0.011, 0.187)	0.080	0.121	(0.021, 0.218)	**0.017**
	9 M	339	−0.034	(−0.140, 0.073)	0.527	0.167	(0.062, 0.269)	**0.002**	0.077	(−0.030, 0.182)	0.156
n6-PUFA	3–6 M	385	0.113	(0.007, 0.217)	**0.026**	0.084	(−0.016, 0.182)	0.101	0.126	(0.026, 0.223)	**0.014**
	9 M	339	−0.038	(−0.144, 0.069)	0.485	0.176	(0.071, 0.277)	**0.001**	0.080	(−0.027, 0.185)	0.143

NEFA, non-esterified fatty acid; M, months; *n*, total number of patients; r, Pearson’s r; HAZ, height-for-age Z score; WAZ, weight-for-age Z score; WHZ, weight-for-height Z score; MUFA, total monounsaturated fatty acids; PUFA, total polyunsaturated fatty acids; n6-PUFA, total n-6 polyunsaturated fatty acids. Significant *p*-values, i.e., at 0.05, are highlighted in bold.

## Data Availability

All generated data are either summarized or represented to support the findings of this work and available within the article.

## Supplementary Materials

The following supporting information can be downloaded at: https://www.mdpi.com/article/10.3390/metabo13040489/s1, Figure S1: Study design; Figure S2: Biosynthesis of monounsaturated and polyunsaturated fatty acids; Figure S3: Correlations between serum NEFAs and serum bile acids in Pakistani children; Figure S4: Heatmaps representing pairwise correlations between all NEFAs (µg/mL of serum) in children with normal (<8 µM) and high (>8 µM) concentrations of total serum bile acids at 3–6 months and 9 months of age; Table S1: Proportions (%) of serum NEFAs in Pakistani undernourished and healthy children at two study time points; Table S2: Correlation between serum NEFA biomarkers (in µg/mL of serum) and growth among Pakistani undernourished and healthy children at between 3–6 months and 9 months of age.
