# Supplementary material for: Essential Fatty Acid Deficiency Associates with Growth Faltering and Environmental Enteric Dysfunction in Children
Source: Metabolites. 2023 Mar 29;13(4):489. doi: 10.3390/metabo13040489 (PMC10142200; doi:10.3390/metabo13040489)
Supplement: Supplementary file 1 [file metabolites-13-00489-s001.zip › metabolites-2233789-supplementary.pdf]

Table S1. Proportions (%) of serum NEFAs in Pakistani undernourished and healthy children at two study time points

	Undernourished children (WHZ<-2 at enrollment)		Healthy control (WHZ>0, HAZ>-1 at enrollment)	
**Age, months**	**3-6 (n=335)**	**9 (n=292)**	**6 (n=50)**	**9 (n=47)**
**SFA**	40.34 ± 0.22	40.12 ± 0.22	40.14 ± 0.51	39.83 ± 0.58
C10:0	0.47 ± 0.02^{B}	0.32 ± 0.01	0.33 ± 0.04	0.3 ± 0.02
C12:0	1.88 ± 0.06^{A}	1.29 ± 0.05	1.34 ± 0.12	1.25 ± 0.1
C14:0	2.98 ± 0.07^{C}	2.57 ± 0.07	2.53 ± 0.16	2.51 ± 0.13
C15:0	0.27 ± 0.01	0.28 ± 0.01	0.26 ± 0.03	0.26 ± 0.03
C16:0	26.35 ± 0.15^{C}	27.35 ± 0.16	27.18 ± 0.37	27.52 ± 0.44
C17:0	0.39 ± 0.01	0.38 ± 0.02	0.41 ± 0.04	0.4 ± 0.05
C18:0	7.73 ± 0.06	7.72 ± 0.06	8.04 ± 0.13	7.51 ± 0.15
C20:0	0.08 ± 0.01^{C}	0.06 ± 0.01	0.05 ± 0.01	0.04 ± 0.01
C22:0	0.1 ± 0.01^{A}	0.06 ± 0.01^{A}	0.02 ± 0.01	0.02 ± 0.01
C24:0	0.06 ± 0.009^{A}	0.015 ± 0.006	0.002 ± 0.002	0.004 ± 0.002
C26:0	0.058 ± 0.012^{A}	0.097 ± 0.012^{A}	0.003 ± 0.003	0.012 ± 0.012
**MUFA**	29.73 ± 0.28^{B}	29.02 ± 0.31	27.71 ± 0.71	28.46 ± 0.5
C14:1n-5	0.05 ± 0.01	0.06 ± 0.02^{C}	0.05 ± 0.02	0.02 ± 0.01
C16:1n-7	2.33 ± 0.05	2.05 ± 0.05	2.1 ± 0.11	2.08 ± 0.12
C17:1n-7	0.51 ± 0.03^{C}	0.41 ± 0.02	0.41 ± 0.03	0.39 ± 0.03
C18:1n-9 cis	24.67 ± 0.24^{C}	24.69 ± 0.27	23.11 ± 0.62	24.01 ± 0.46
C18:1n-7	1.9 ± 0.05	1.68 ± 0.04	1.92 ± 0.11	1.84 ± 0.14
C20:1n-9	0.31 ± 0.07^{C}	0.15 ± 0.03	0.13 ± 0.03	0.12 ± 0.06
**n3-PUFA**	3.14 ± 0.07	3.16 ± 0.09	2.94 ± 0.15	2.88 ± 0.14
C18:3n-3	0.75 ± 0.03	0.78 ± 0.04	0.67 ± 0.07	0.86 ± 0.11
C20:3n-3	0.063 ± 0.011^{A}	0.127 ± 0.037^{B}	0.013 ± 0.004	0.004 ± 0.002
C20:5n-3	0.07 ± 0.006	0.096 ± 0.01	0.064 ± 0.015	0.056 ± 0.014
C22:3n-3	0.95 ± 0.05	0.79 ± 0.05	0.78 ± 0.12	0.63 ± 0.09
C22:5n-3	0.25 ± 0.01	0.28 ± 0.01	0.3 ± 0.03	0.27 ± 0.02
C22:6n-3	1.06 ± 0.02	1.09 ± 0.03	1.12 ± 0.06	1.06 ± 0.04
**n6-PUFA**	26.82 ± 0.29^{B}	27.71 ± 0.34	29.24 ± 0.93	28.85 ± 0.72
C18:2n-6 cis	20.23 ± 0.24^{B}	21.52 ± 0.28	22.33 ± 0.75	22.65 ± 0.64
C18:3n-6	0.12 ± 0.02	0.12 ± 0.01	0.09 ± 0.02	0.07 ± 0.03
C20:2n-6	0.13 ± 0.01	0.1 ± 0.01^{B}	0.09 ± 0.01	0.05 ± 0.01
C20:3n-6	0.88 ± 0.03	0.83 ± 0.02	0.93 ± 0.05	0.79 ± 0.05
C20:4n-6	5.04 ± 0.08	4.76 ± 0.1	5.42 ± 0.25	4.95 ± 0.18
C22:2n-6	0.006 ± 0.003	0.012 ± 0.004	0.016 ± 0.011	0.006 ± 0.006
C22:4n-6	0.13 ± 0.01	0.12 ± 0.01	0.11 ± 0.01	0.07 ± 0.01

C22:5n-6	0.29 ± 0.01	0.26 ± 0.01	0.25 ± 0.02	0.24 ± 0.02

Data are represented as mean and SEM. Difference in each characteristic were evaluated by corresponding t-test. [A]*P*<0.0001 compared with the healthy control group at the corresponding time point. [B]*P*<0.01 compared with the healthy control group at the corresponding time point. [C]*P*<0.05 compared with the healthy control group at the corresponding time point. SFA, total saturated fatty acids; MUFA, total monounsaturated fatty acids; n3-PUFA, total n-3 polyunsaturated fatty acids; n6-PUFA, total n-6 polyunsaturated fatty acids.

Table S2. Correlation between serum NEFA biomarkers (in μg/mL of serum) and growth among Pakistani undernourished and healthy children at between 3 and 6 months and 9 months of age.

NEFA	Age	Total no.	WHZ			*HAZ*			*WAZ*		
			Pearson's r	Coefficient range (95% CI)	*P*-Value	Pearson's r	Coefficient range (95% CI)	*P*-Value	Pearson's r	Coefficient range (95% CI)	*P*-Value
**C10:0**	3-6 months	385	-0.138	(-0.235, -0.039)	**0.007**	-0.069	(-0.168, 0.031)	0.176	-0.121	(-0.218, -0.021)	**0.018**
	9 months	339	0.082	(-0.024, 0.187)	0.130	0.068	(-0.038, 0.174)	0.209	0.083	(-0.024, 0.188)	0.126
**C12:0**	3-6 months	385	-0.110	(-0.208, -0.011)	**0.030**	-0.127	(-0.224, -0.027)	**0.013**	-0.144	(-0.241, -0.045)	**0.005**
	9 months	339	0.004	(-0.103, 0.110)	0.948	0.036	(-0.071, 0.142)	0.508	0.013	(-0.094, 0.119)	0.810
**C14:0**	3-6 months	385	-0.065	(-0.164, 0.035)	0.205	-0.110	(-0.208, -0.011)	**0.030**	-0.106	(-0.204, -0.006)	**0.037**
	9 months	339	0.028	(-0.079, 0.134)	0.604	0.009	(-0.098, 0.116)	0.867	0.015	(-0.092, 0.121)	0.784
**C18:1n-7**	3-6 months	385	-0.018	(-0.118, 0.082)	0.719	-0.044	(-0.143, 0.056)	0.387	-0.043	(-0.142, 0.057)	0.403
	9 months	339	0.009	(-0.097, 0.116)	0.866	-0.119	(-0.223, -0.013)	**0.028**	-0.074	(-0.179, 0.033)	0.172
**C18:2n-6 cis**	3-6 months	385	0.072	(-0.028, 0.171)	0.160	0.024	(-0.076, 0.124)	0.632	0.060	(-0.040, 0.159)	0.244
	9 months	339	-0.043	(-0.149, 0.064)	0.426	0.118	(0.012, 0.222)	**0.029**	0.036	(-0.070, 0.142)	0.505
**C18:3n-6**	3-6 months	385	-0.029	(-0.129, 0.071)	0.576	-0.134	(-0.231, -0.035)	**0.008**	-0.117	(-0.214, -0.017)	**0.021**
	9	339	-0.003	(-0.110, 0.103)	0.950	0.037	(-0.070, 0.143)	0.502	0.013	(-0.094, 0.119)	0.810

	months										
**C20:1n-9**	3-6 months	385	-0.069	(-0.168, 0.031)	0.177	-0.006	(-0.1059, 0.0940)	0.901	-0.043	(-0.142, 0.057)	0.396
	9 months	339	0.143	(0.037, 0.246)	**0.008**	-0.009	(-0.116, 0.097)	0.866	0.078	(-0.029, 0.183)	0.152
**C20:3n-3**	3-6 months	385	-0.073	(-0.172, 0.027)	0.154	-0.104	(-0.202, -0.004)	**0.042**	-0.105	(-0.203, -0.005)	**0.040**
	9 months	339	-0.060	(-0.165, 0.047)	0.274	-0.078	(-0.183, 0.029)	0.153	-0.074	(-0.179, 0.033)	0.176
**C20:4n-6**	3-6 months	385	0.047	(-0.053, 0.146)	0.354	0.118	(0.018, 0.215)	**0.020**	0.110	(0.010, 0.208)	**0.031**
	9 months	339	-0.041	(-0.146, 0.066)	0.457	0.155	(0.050, 0.258)	**0.004**	0.065	(-0.042, 0.170)	0.232
**C22:6n-3**	3-6 months	385	0.020	(-0.080, 0.120)	0.702	0.176	(0.077, 0.271)	**0.001**	0.141	(0.042, 0.238)	**0.006**
	9 months	339	-0.038	(-0.144, 0.069)	0.483	0.168	(0.062, 0.269)	**0.002**	0.073	(-0.033, 0.179)	0.178
**C24:0**	3-6 months	385	-0.084	(-0.182, 0.016)	0.101	-0.110	(-0.208, -0.010)	**0.031**	-0.121	(-0.218, -0.021)	**0.017**
	9 months	339	0.048	(-0.059, 0.153)	0.382	0.078	(-0.029, 0.183)	0.154	0.074	(-0.033, 0.179)	0.172
**Total PUFA**	3-6 months	385	0.041	(-0.059, 0.140)	0.425	0.033	(-0.067, 0.133)	0.517	0.049	(-0.051, 0.148)	0.340
	9 months	339	-0.037	(-0.143, 0.069)	0.493	0.120	(0.014, 0.224)	**0.027**	0.042	(-0.065, 0.148)	0.441
**n6-PUFA**	3-6 months	385	0.063	(-0.037, 0.162)	0.220	0.036	(-0.064, 0.136)	0.477	0.062	(-0.038, 0.161)	0.222
	9 months	339	-0.041	(-0.147, 0.066)	0.453	0.130	(0.024, 0.233)	**0.017**	0.046	(-0.061, 0.152)	0.402

HAZ, height-for-age Z score; WAZ, weight-for-age Z score; WHZ, weight-for-height Z score; PUFA, total polyunsaturated fatty acids; n6-PUFA, total n-6 polyunsaturated fatty acids.

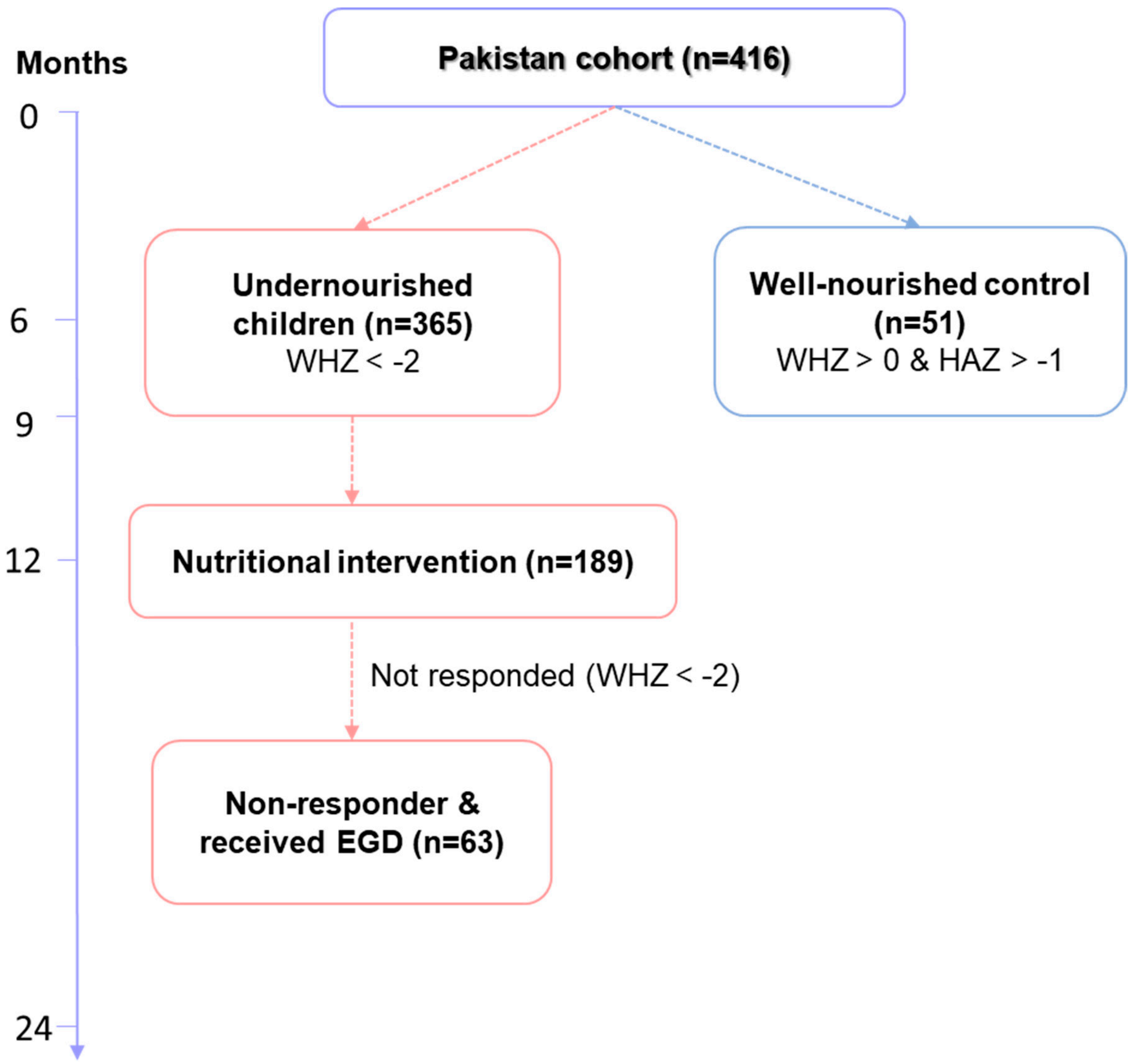

Figure S1. Study design.

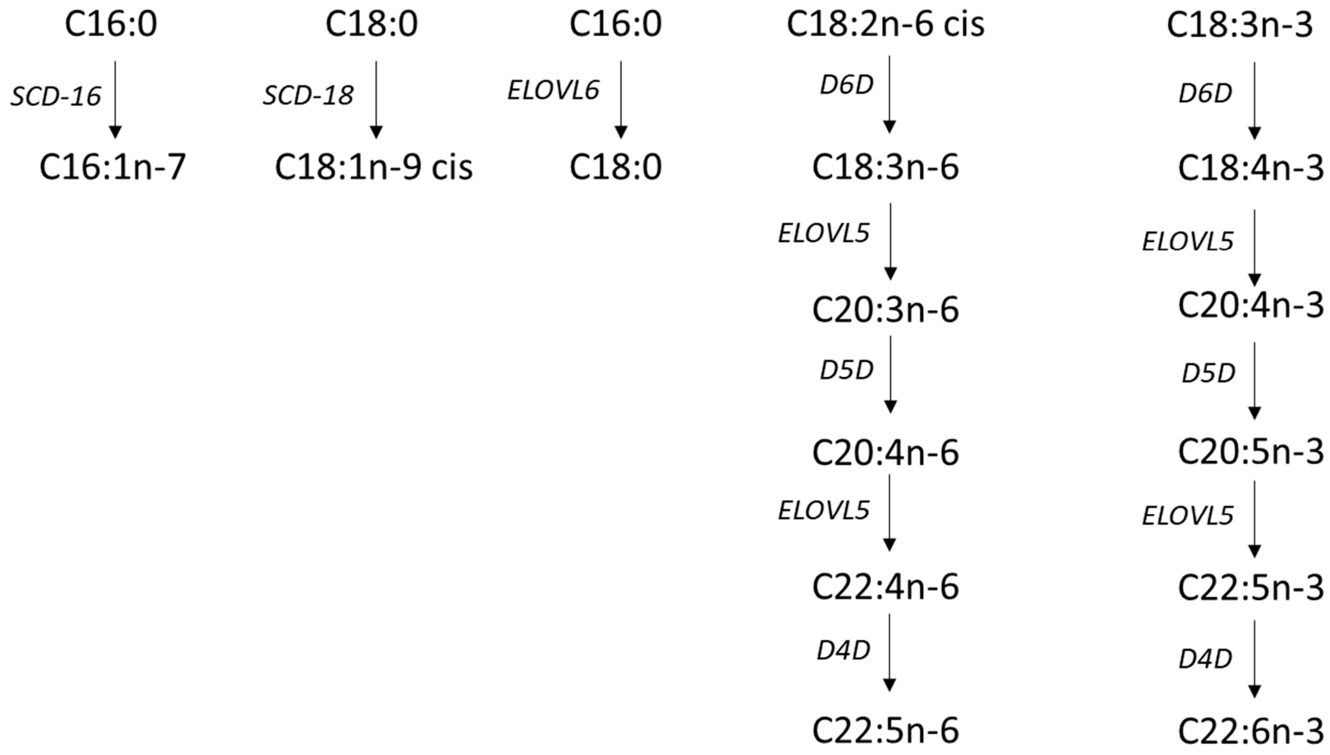

Figure S2. Biosynthesis of monounsaturated and polyunsaturated fatty acids. SCD: stearoyl-CoA desaturase, D4D: delta-4-desaturase, D5D: delta-5-desaturase, D6D: delta-6-desaturase, D8D: delta-8-desaturase, ELOVL5: elongase 5, ELOVL6: elongase 6.

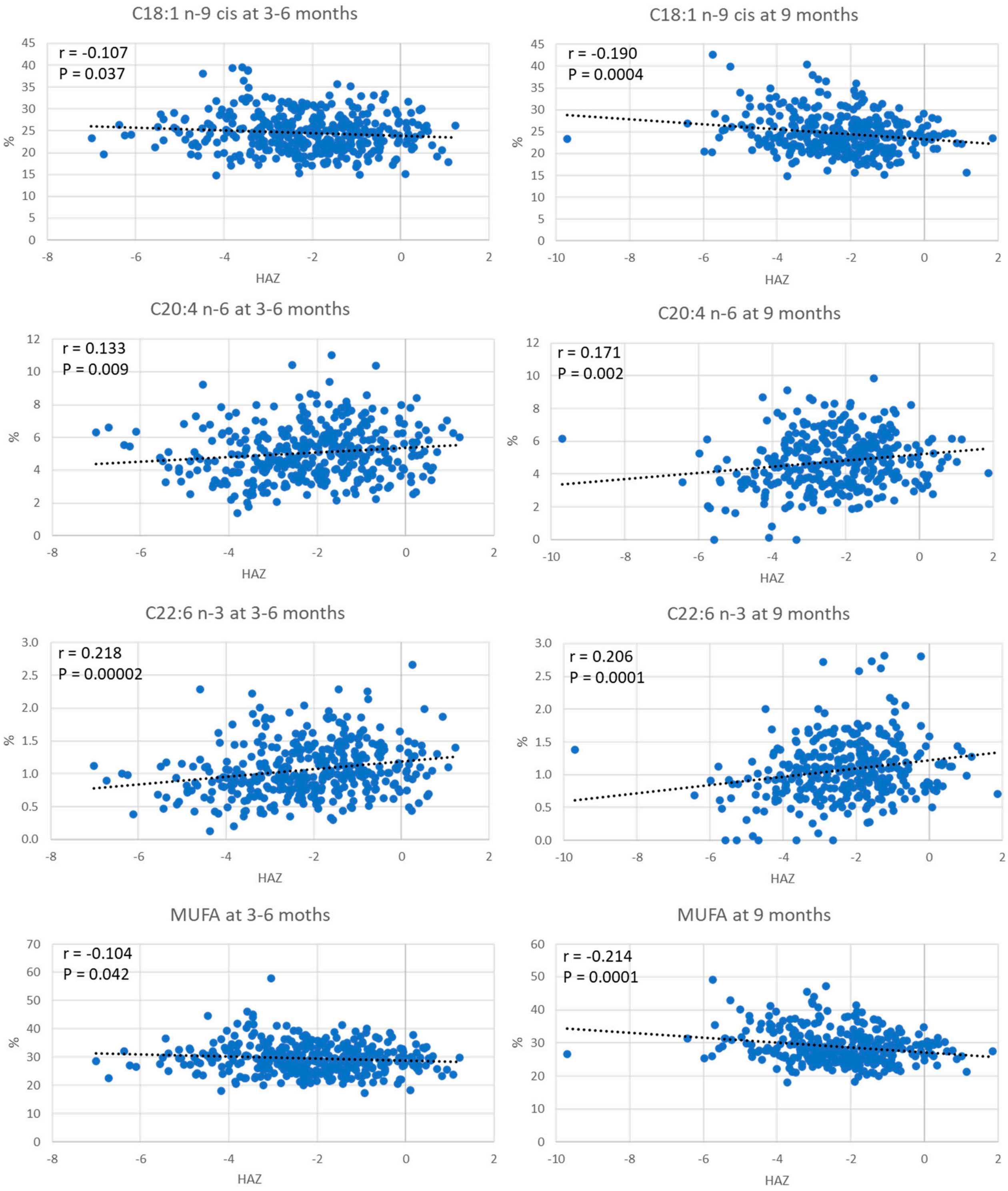

Figure S3. Significant correlation between serum non-esterified fatty acid biomarkers (expressed as % composi-tion) and height-for-age Z score (HAZ) among Pakistani undernourished and healthy children at between 3-6 months and 9 months of age.

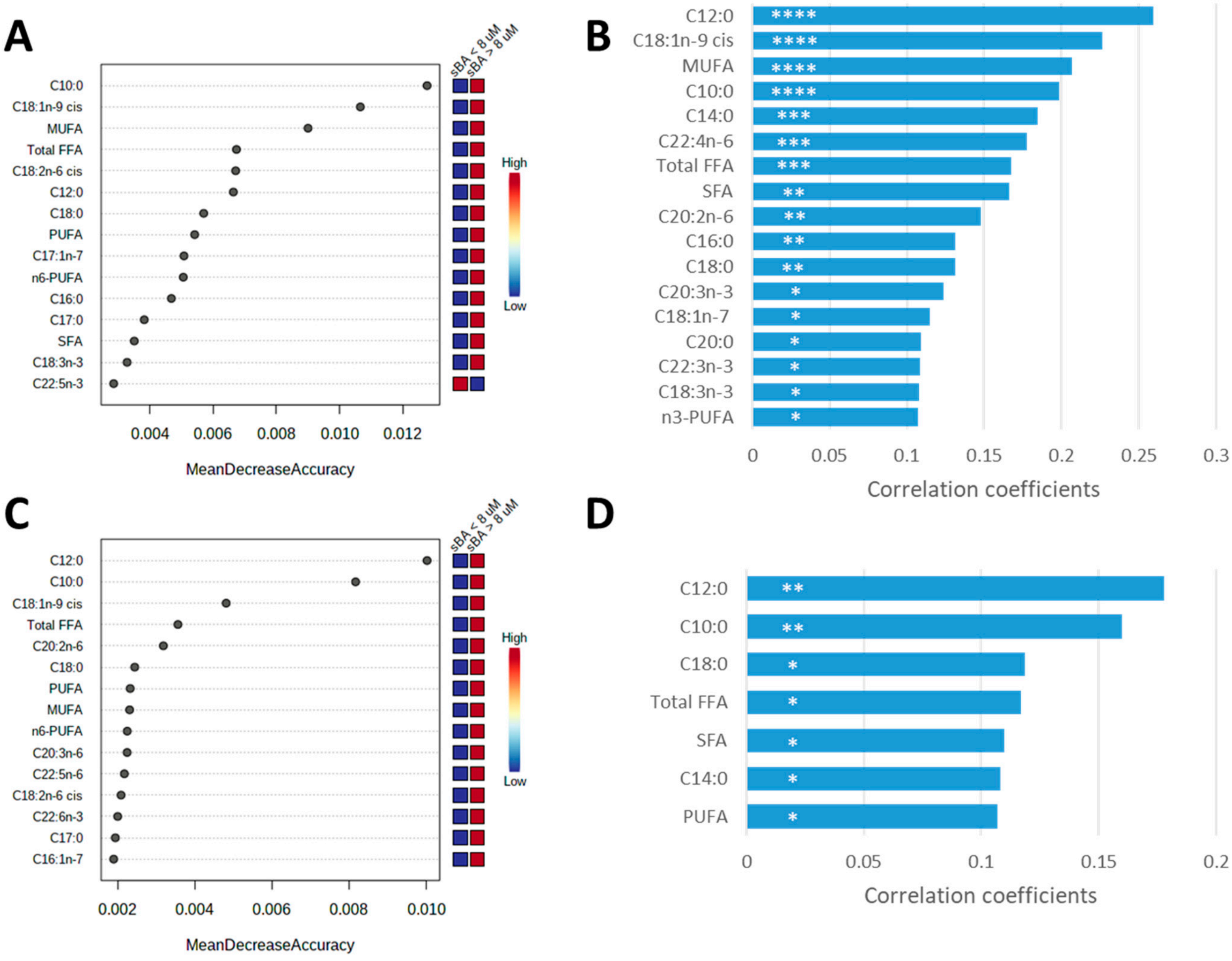

Figure S4. Correlations between serum NEFAs and serum bile acids in Pakistani children. Top 15 significant NEFAs calculated by Random Forest for classification accuracy between Pakistani children with normal and high values of total sBA in at the age of 3-6 (A) and 9 (C) months. Concentrations of NEFAs were correlated with sBA at 3-6 (B) and 9 (D) months of age using a pattern search and Pearson's r as distant measure. Significant linear correlations are considered when p-value is lower than 0.05. **P*<0.05, ***P*<0.01, ****P*<0.001, *****P*<0.0001.

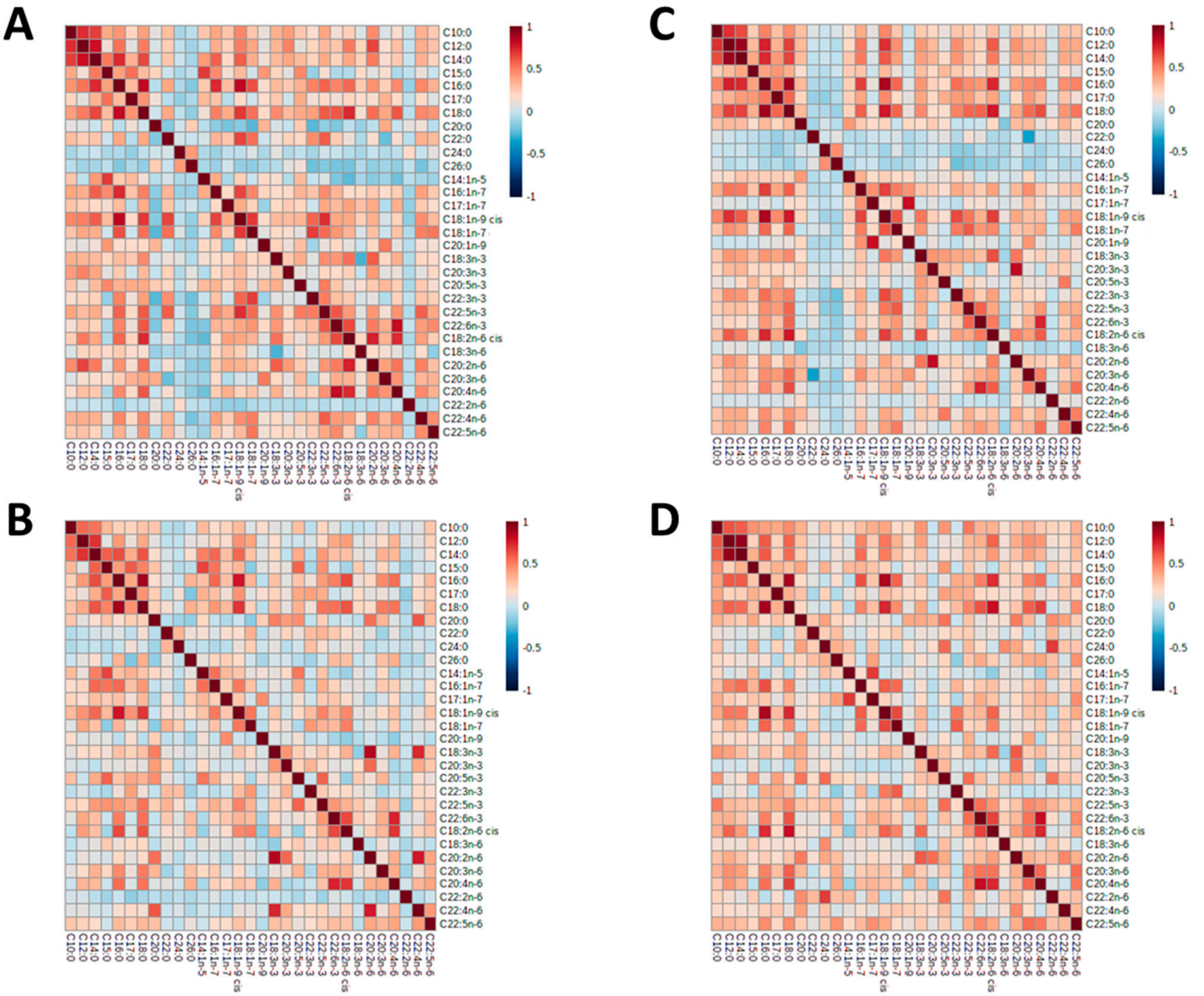

Figure S5. Heatmaps representing pairwise correlations between all NEFA (µg/mL of serum) in children with normal (< 8 µM) and high (> 8 µM) concentrations of total serum bile acids at 3-6 months and 9 months of age. In the heatmap, red represents higher correlation, and blue shows lower correlation between a pair of markers. (A) Children with sBA concentrations < 8 µM at 3-6 months. (B) Children with sBA concentrations > 8 µM at 3-6 months. (C) Children with sBA concentrations < 8 µM at 9 months. (D) Children with sBA concentrations > 8 µM at 9 months. sBA, serum bile acids.
